# Lipopolysaccharide-Activated Bone Marrow-Derived Dendritic Cells Suppress Allergic Airway Inflammation by Ameliorating the Immune Microenvironment

**DOI:** 10.3389/fimmu.2021.595369

**Published:** 2021-05-19

**Authors:** Zhihui Min, Yuzhen Zeng, Tao Zhu, Bo Cui, Ruolin Mao, Meiling Jin, Zhihong Chen

**Affiliations:** ^1^Research Center of Zhongshan Hospital, Fudan University, Shanghai, China; ^2^Department of Pulmonary and Critical Care Medicine, Shanghai Institute of Respiratory Disease, Zhongshan Hospital, Fudan University, Shanghai, China; ^3^Department of Respiratory Medicine, Second Affiliated Hospital of Chongqing Medical University, Chongqing, China

**Keywords:** asthma, lipopolysaccharide, bone marrow-derived dendritic cells, memory CD4^+^ T cell, adoptive transfer

## Abstract

**Background:**

Previous studies have shown that lipopolysaccharide (LPS)-activated bone marrow-derived dendritic cells (DClps) might induce tolerance in autoimmune and cancer models *in vivo*, whereas it remains unclear whether DClps could play a role in allergic disease model. Herein, we aimed to elucidate the potential effects of DClps on OVA-sensitized/challenged airway inflammation in a mouse model, which may help facilitate the application of specific tolerogenic dendritic cells (tolDC) in allergic asthma in the future.

**Methods:**

The phenotype and function of immature DC (DCia), DClps or IL-10-activated-DC (DC10) were determined. OVA-sensitized/challenged mice were treated with OVA-pulsed DCia or DClps or DC10. We assessed the changes of histopathology, serum total IgE level, pulmonary signal transducers and activators of transcription (STAT), pulmonary regulatory T cells (Tregs), and airway recall responses to OVA rechallenge, including proliferation and cytokine secretory function of pulmonary memory CD4^+^ T cells in the treated mice.

**Results:**

DClps exhibited low levels of CD80 and MHCII and increased levels of anti-inflammatory cytokines such as IL-10 and TGF-β. Additionally, DClps treatment dramatically diminished infiltration of inflammatory cells, eosinophilia, serum IgE and STAT6 phosphorylation level, increased the number of pulmonary Tregs. In addition, DClps treatment decreased the proliferation of pulmonary memory CD4^+^ T cells, which further rendered the downregulation of Th2 cytokines *in vitro*.

**Conclusion:**

LPS stimulation may lead to a tolerogenic phenotype on DC, and thereby alleviated the Th2 immune response of asthmatic mice, possibly by secreting anti-inflammatory cytokines, inhibiting pulmonary memory CD4^+^ T cells, downregulating pulmonary STAT6 phosphorylation level and increasing pulmonary Tregs.

## Introduction

Dendritic cells (DCs) are critically involved in the modulation of Th1 and Th2 immune responses, and induction of Tregs to mediate tolerance (1). Physiologically, immature DCs (DCia) are distributed in peripheral tissues, while mature DCs with higher expression level of MHCII, CD80 and CD86 can elicit adaptive immune responses (2). Nevertheless, DCs may present distinct phenotypic and functional characteristics according to the stimulus that they received. Ample evidence has demonstrated that DCs differentiated under certain conditions are capable of establishing immune tolerance in multiple disease models (3–5). These specific tolDCs are distinguished by the decreased expression of MHCII, CD80 and CD86 and proinflammatory cytokines IL-12 along with enhancement of anti-inflammatory cytokines IL-10 and TGF-β, thus leading to T cell anergy and Treg activation (6).

A large number of *in vitro* protocols have been established to generate tolDCs that exert prophylactic impacts on Th1-mediated immunopathogenesis, whereas how Th2-mediated allergic reactions are modulated remains obscure (7, 8). IL-10, an anti-inflammatory and immunoregulatory cytokine, was confirmed to be a more potent inducer of tolDCs than vitamin D (3), dexamethasone, TGF-β and rapamycin (9, 10). IL-10-induced DCs with low expression of MHCII and costimulatory molecules but high secretion of IL-10 can lead to Tregs activation (11). Furthermore, it has been reported that IL-210-activated DCs can abrogate experimental asthma (12), while several studies have recently demonstrated that LPS, which is most frequently used to stimulate DCs maturation *in vitro* to initiate immune responses, can also render DCs to exert immunosuppressive effects by producing sufficient IL-10 (13, 14). For example, it was found that LPS pretreatment modified the phenotype of DCs, thus blocking experimental autoimmune encephalomyelitis (EAE) development by suppressing effector CD4^+^ T cells (13). Additionally, it is proved that although LPS-activated DC maturation may lead to DCs apoptosis, DCia can immediately take up apoptotic DCs and then convert into tolDCs that induce augmented TGF-β secretion and Foxp3^+^ Treg differentiation (15). Taken together, these findings suggest that LPS might play different roles in modulating the phenotypes and functions of DCs under various conditions. As asthma is characterized with complex immune microenvironment, whether LPS confers tolerogenic capabilities in DCs and the potential intervention effects on allergic airway inflammation warrant further investigation.

Herein, the study critically aims to evaluate the impact of the intraperitoneal adoptive transfer of DClps on airway inflammation and pulmonary memory CD4^+^ T cells, Tregs, and STAT expression in asthmatic models.

## Materials and Methods

### Mice

Six‐week‐old female C57BL/6 mice were obtained from Silaike Experimental Animal Limited Liability Company (Shanghai, China). All animals were treated in accordance with the National Institutes of Health Guidelines and Regulations.

### Isolation and Characterization of BMDCs

BMDCs were generated as noted (12, 16). Briefly, BM cells were seeded in RPMI-1640 supplemented with 1% antibiotics/antimycotics and 10% heat-inactivated fetal calf serum (FCS) containing 20 ng/ml GM-CSF. A fresh complete medium change was performed on days 3 and 6. On day 8, nonadherent DCs were collected and resuspended in complete medium supplemented with PBS (to generate DCia) or 50 ng/ml IL-10 or 10 ng/ml LPS (to generate DC10, DClps) for 24 h. On day 9, cells were pulsed for 2 h at 37°C with 1 μM OVA (Sigma-Aldrich, Grade V; St. Louis, MO, USA) and then the cells were harvested (**Supplementary Figure 1A**). Cells were determined by flow cytometry using APC-Cy7-CD11c, APC-CD80, PE-CD86 and FITC-MHCII specific antibodies (eBioscience, Inc., San Diego, CA, USA) and were subsequently analyzed (FCM Canto II; BD Biosciences, San Jose, CA, USA). All data were analyzed with Flow Jo software.

### Establishment of OVA-Induced Asthmatic Mice and the Adoptive Transfer of DCs

To establish an asthma phenotype, 6-week-old female C57BL/6 mice were intraperitoneally sensitized with PBS containing 100 µg OVA in 2 mg alum or PBS/alum as a control on days 0 and 7. Animals were then intranasally challenged with 100 μg OVA or PBS as a control under anesthetic on days 14–18. For the treatment models, 1 × 10^6^ cells (DCia, DC10 or DClps prepared as described above) were intraperitoneally transferred on days 14–16.

At 24 h after the last challenge, mice were anesthetized, and bronchoalveolar lavage (BAL) was performed with cold PBS. Subsequently, lungs were obtained for histology and fixed with 4% paraformaldehyde in PBS. BAL fluid (BALF) was centrifuged (1,200 rpm for 10 min, at 4°C), and cells were obtained to assess the total cell number and differential cell counts. Cells sedimented by centrifugation were dissolved with ACK Lysis Buffer, and then the total cells in BALF were counted. Wright–Giemsa stain was used for the differential cell count in BALF. Each sample was counted for three times.

### Sorting of Pulmonary Memory CD4^+^ T Cells From OVA-Induced Asthmatic Mice

Another asthma model was established in mice to separate pulmonary memory CD4**^+^** T cells. The OVA sensitization and challenge protocol was similar to the previous protocol. DCia, DC10 or DClps were transferred to OVA-sensitized mice for 3 consecutive days (days 14–16), respectively. However, the mice were not sacrificed on day 19, but re-challenged with OVA for 5 consecutive days (days 35–39) and sacrificed on day 40 for the separation of pulmonary memory T cells. Lung mononuclear cell suspensions were generated, and then determined as memory T cells labeling with CD4 and CD44 by FCM (**Supplementary Figure 2**). Finally, CD4**^+^**CD44**^+^** T cells were sorted by fluorescence-activated cell sorting (FACS). Effector memory CD4**^+^** T cells (T_EM_) and central memory CD4**^+^** T cells (T_CM_) were determined by evaluating the expression of CD4, CD44, CD62L and CCR7, and then the two cell subpopulation labeled with CD4, CD44, CD62 and CCR7 specific antibodies were sorted by FACS. All data were analyzed with Flow Jo software.

### Mixed Lymphocyte Reaction (MLR) of Pulmonary Memory CD4^+^ T Cells and BMDCs

Pulmonary memory CD4**^+^**CD44^+^ T cells, T_CM_ or T_EM_ cells and BMDCs were obtained as described above. BMDCs were pretreated with culture medium containing 200 μg/ml mitomycin C for 20 min at 37°C (Kyowa Hakko Kogyo, Tokyo, Japan). Pulmonary memory CD4**^+^**CD44^+^ T cells were labeled with carboxyfluorescein diacetate succinimidyl ester (CFSE, Invitrogen Ltd., UK) prior to culture to evaluate T cell proliferation. Mitomycin C-treated BMDCs (l × 10^5^/well) and memory CD4**^+^**CD44^+^ T cells (4 × l0^5^/well) were cocultured in 96-well plates with 50 ug/ml OVA at a ratio of 1:4 at 37°C, 5% CO_2_ atmosphere for 1, 3 or 5 days, respectively. The co-culture systems were divided into five groups: the control group, the OVA group, the OVA + DCia group, the OVA + DC10 group and the OVA + DClps group. After MLR assay, the proliferation activity of memory CD4^+^CD44^+^ T cells was evaluated by FCM on days 1, 3, and 5, respectively. T_CM_ or T_EM_ cells were co-cultured with BMDCs to perform MLR assay, respectively. On day 5, cells in the co-culture systems were stimulated with phorbol myristate acetate (PMA; 100 ng/ml, Sigma) and Inomycin (5 µmol/L, Sigma) overnight, and then cell supernatants were collected. IL-4 and IFN-γ level in the cell supernatants was determined by ELISA.

### Pulmonary Tregs of OVA-Induced Asthmatic Mice

As noted, lung tissues of asthmatic mice were immediately collected on day 19 to determine pulmonary Tregs. Single-cell suspensions of lung parenchymal cells were generated by the enzymatic digestion of tissues, and Tregs were characterized as CD4**^+^**CD25**^+^**Foxp3**^+^** cells by FCM. A total of 1 × 10^6^ cells were washed and stained with FITC-conjugated anti-CD4, PE-conjugated anti-CD25 and APC-conjugated anti-Foxp3 (eBioscience) antidodies, and subsequently analyzed by FCM (**Supplementary Figure 3**). All data were analyzed with Flow Jo software.

### Tracking BMDC in the Lung

We labeled transferred DC with PKH26 fluorescent dye (Sigma‐Aldrich) according to the instructions. Briefly, we used 1 ml dilution buffer to suspend 2 × 10^6^ DC and then mixed the cell suspension with the same amount of labeling solution containing 4 × 10^−6^ M PKH26 dye in dilution buffer. After 4 min of incubation at room temperature, we added 2 ml fetal bovine serum (FBS) to terminate the reaction and washed the cells with a control buffer. Finally, 5 × 10^6^ DC labeled with PKH26 dye was mixed in 1 ml PLA‐CMC solution for the subsequent adoptive transfer. PKH26-labeled DC were intraperitoneally injected into asthmatic mice (1 × 10^6^ cells/mouse), as described above. After 1, 3 and 7 days, mice were sacrificed respectively, and lung tissues from each animal were removed. DCs labeling with PKH26 were determined by separating lung mononuclear cells, and then cells were stained with MHCII antibodies to eliminate the interference of other cell types. The transferred DCs in the lung were assessed *via* the expression of MHCII and PKH26 as determined by FCM. All data were analyzed with Flow Jo software.

### Histopathologic Analysis of the Lung

The left lung was removed from each mouse and infused with 4% paraformaldehyde after BAL, embedded in paraffin, sectioned at 5 μm, and stained with hematoxylin and eosin (H&E) according to standard procedures for evaluating histopathological changes in bronchial and lung tissues. Masson stain of the lung was also performed to observed collagen fibers (**Supplementary Figure 4**). Right lung specimens were collected and frozen at −80°C for protein analysis by Western blotting (WB).

Peribronchial and perivascular inflammation were estimated with a subjective scale of 0–3 based on a scoring system: a score of 0 indicated that there was no detectable inflammation; 1 indicated that most bronchi or vessels were surrounded by a thin layer (one to five cells thick) of inflammatory cells; 2 indicated that most bronchi were surrounded by a thin layer (one to five cells) of inflammatory cells; 3 indicated that most bronchi or vessels were surrounded by a thick layer (>5 cells) of inflammatory cells (17). The average peribronchial and perivascular inflammation scores reflected the total lung inflammation of treated animals. The score is presented as a mean value of each animal.

### Cytokine Releasing Capacity of BMDC

BMDC (DCia, DC10, and DClps) were harvested after 9 days of culture, as described above. Cells from each culture were pulsed for 2 h at 37 °C with 1 μM OVA. The cell culture supernatant was collected and stored at −80°C for subsequent cytokine detection of IL-10, TGF-β, MCP-3, IL-12 and IFN-γ by ELISA (R&D Systems).

### WB Analysis

Expression levels of STAT1, 4, and 6 and their phosphorylated protein levels in lung digests were assessed by WB. Total protein from mouse right lung tissue was extracted by using RIPA Protein Extracted Reagent (Thermo). 40 μg lysate samples were fractionated by 10% sodium dodecyl sulfate (SDS)-PAGE and transferred to polyvinylidene fluoride (PVDF) membranes (Roche, USA). After incubation with blocking buffer containing 5% skim milk in TBST (12.5 mM Tris–HCl pH 7.5, 68.5 mM NaCl, 0.1% Tween 20) for 1 h, the membrane was then incubated with primary antibodies overnight, including rabbit anti-STAT1, and rabbit anti-phosphorylated STAT1, rabbit anti-STAT4, rabbit anti-phosphorylated STAT4, rabbit anti-STAT6, rabbit anti-phosphorylated STAT6 antibodies. The anti-mouse GAPDH antibody was used as a loading control. The membrane was washed with TBST and incubated with horseradish-peroxidase-conjugated secondary antibody (Jackson ImmunoResearch) and then developed with and electrochemiluminescence (ECL) substrate solution (Millipore). The membrane was incubated with rabbit anti-murine GAPDH for assessment of internal protein loading control after incubation with stripping buffer for 15 min at 25°C. The density of each band was quantified using image analysis software (ImageJ).

### Statistical Analysis

All data are representative of at least three independent experiments. Each error bar represents the standard deviation (SD). Multigroup comparisons were assessed by either one-way ANOVA with Tukey’s *post hoc* test or by Wilcoxon signed rank tests (FCM analyses) with a commercial software program (GraphPad Prism 8.0, San Diego, CA). P <0.05 was considered statistically significant.

## Results

### Secretory Capacity and Costimulatory Molecule Expression of DCs

**Figure 1:**
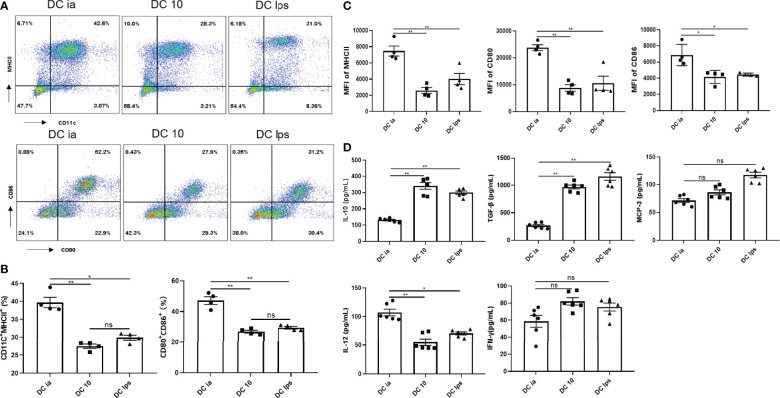
Secretory capacity and costimulatory molecule expression of DCs. **(A)** The percentage of CD11c^+^MHCII^+^ cells, and CD80^+^CD86^+^ expression on CD11c^+^MHCII^+^ cells determined by FCM. **(B)** Percentage of CD11c^+^MHCII^+^ and CD11c^+^MHCII^+^CD80^+^CD86^+^ cells determined by FCM. **(C)** MFI of MHCII, CD80 and CD86 determined by FCM. **(D)** The levels of IL-10, TGF-β, MCP-3, IL-12 and IFN-γ secreted by DCia, DC10 and DClps were determined by ELISA. The columns and error bars represent the mean and standard error of the mean (SEM). (*P < 0.05, **P < 0.01, ns, no significant difference, ANOVA with Tukey’s post hoc analysis). Data are representative of 3 independent experiments with similar results (n=5 in each group).

BMDC labeled with APC-Cy7-CD11c, APC-CD80, PE-CD86, and FITC-MHCII specific antibodies were determined by flow cytometry (FCM). As shown in the results, BM cells were triple negative for CD11c, CD80, CD86 and MHCII, while BMDC were triple positive for those markers (**Supplementary Figure 1B**). Compared with DCia, the proportion of CD11c^+^MHCII^+^ cells and CD11c^+^MHCII^+^CD80^+^CD86^+^ cells and in DC10 and DClps was lower (**Figures 1A, B**), the same results were shown in MFI of MHCII, CD80, CD86 (**Figure 1C**).

In addition, the secretion of certain inflammatory cytokines (IL-10, TGF-β, IL-12 and IFN-γ) is considered essential for characterizing the tolerogenic potential of BMDC. BMDC expressed CCR1, CCR2, and CCR3, all of which bind MCP-3 (18). Thus, we collected BMDC supernatant and examined IL-10, TGF-β, MCP-3, IL-12 and IFN-γ. Both DC10 and DClps secreted high level of IL-10 and TGF-β but low level of IL-12 (**Figure 1D**). DCia secreted lower level of IL-10 and TGF-β but higer level of IL-12 (**Figure 1D**). No distinct differences were found in the secretion of MCP-3 or IFN-γ among cells (**Figure 1D**).

### Adoptive Transfer of DClps Alleviated Airway Inflammation in OVA-Induced Asthmatic Mice

**Figure 2:**
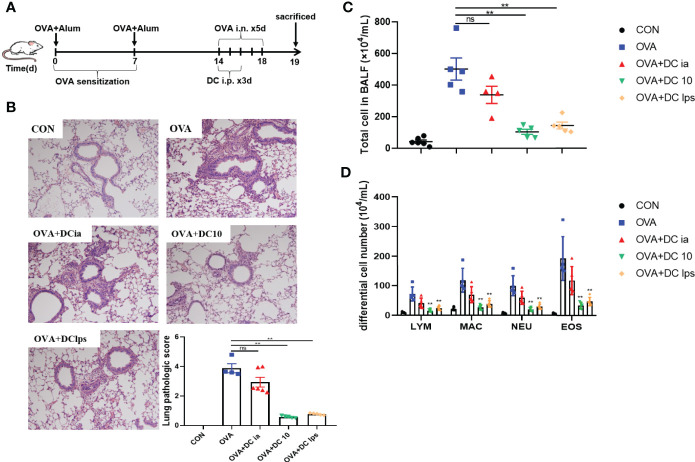
Adoptive transfer of DClps alleviated airway inflammation in OVA-induced asthmatic mice. **(A)** An asthma model was induced in C57BL/6 mice by OVA-sensitization/challenge. DCia, DC10 or DClps were transperitoneally transferred to OVA-sensitized mice. **(B)** Representative H&E stain of lung (100×) and lung pathologic score. **(C, D)** Total and differential cell in BALF. The columns and error bars represent the mean and SEM. (**P < 0.01, ns, no significant difference, compared with the OVA group, ANOVA with Tukey’s *post hoc* analysis). The same experiment was repeated three times with similar results (n = 5 in each group).

We used OVA sensitization/challenge to establish an asthma mouse model (**Figure 2A**). In this experiment, BMDC were generated *in vitro* and used to treat asthmatic mice, as noted. After OVA stimulation, we found that the lung tissue of asthmatic mice exhibited typical inflammatory changes, which were characterized by airway wall thickening, and inflammatory cell infiltration (**Figure 2B**). Additionally, the OVA group showed a remarkable increase of eosinophils in BALF (**Figure 2D**). DClps treatment markedly improved the lung inflammation (**Figure 2B**), as well as dramatically diminished total inflammatory cells and eosinophils in BALF (**Figures 2C, D****)**. The analysis of differential cells in BALF suggesting that adoptive transfer of DClps may be involved in attenuating lung eosinophilic inflammation in asthmatic mice.

### Adoptive Transfer of DClps Effectively Migrated to the Lung of OVA-Induced Asthmatic Mice

**Figure 3:**
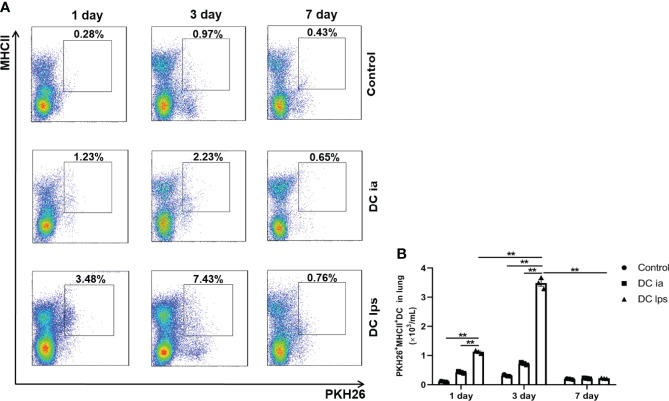
Adoptive transfer of DClps effectively migrated to the lung of OVA-induced asthmatic mice. **(A)** The transferred DCs were stain with PKH26 fluorescent dye. Representative FCM dot plots of PKH26^+^MHCII^+^ DC on days 1, 3 and 7 are shown. **(B)** Numbers of PKH26^+^MHCII^+^ DC in the total lung mononuclear cells. The columns and error bars represent the mean and SEM. (**P < 0.01, ANOVA with Tukey’s *post hoc* analysis). The same experiment was repeated three times with similar results (n = 3 in each group).

The function of DCs is intimately associated with their capacity to migrate to target organs (19). It was previously demonstrated that the onset of tolerance in DC10-treated asthmatic mice is progressive; thus, airway hyperresponsiveness (AHR) in mice is mildly attenuated at 2 weeks after transfer and vanishes entirely within 3 weeks (20). To examine when and whether the injected DClps could successfully migrate to the lungs, we established a mouse model of asthma and treated asthma model mice with DCia, DClps or DC10. We used the fluorescent dye PKH26 as a marker for trafficking the transferred DCs *in vivo* in treated mice, and mice were sacrificed on days 1, 3, and 7 (**Figure 3**). One day after transfer, PKH26^+^MHCII^+^ DC in DClps group appeared in the lungs with a modest number. Nevertheless, the number of PKH26^+^MHCII^+^ DC in DClps group was higher than that in DCia group (p <0.01). On day 3, the number of PKH26^+^MHCII^+^ DC peaked in the lungs, among which DClps group increased the most markedly (p <0.01). However, on day 7, the number of PKH26^+^MHCII^+^ DC strikingly decreased in DClps group and no detectable difference was found among the groups (p >0.05). These data indicate that DClps migrated to the lungs more effectively and gradually accumulated in the lungs within 1 week. Moreover, the decreased of DClps in the lungs was consistent with the alleviation of lung inflammation in asthmatic mice, which further confirmed that DClps may effectively migrate to the lungs and take effect within 1 week.

### Adoptive Transfer of DClps Reduced the Number of Pulmonary Memory CD4^+^ T Cells

**Figure 4:**
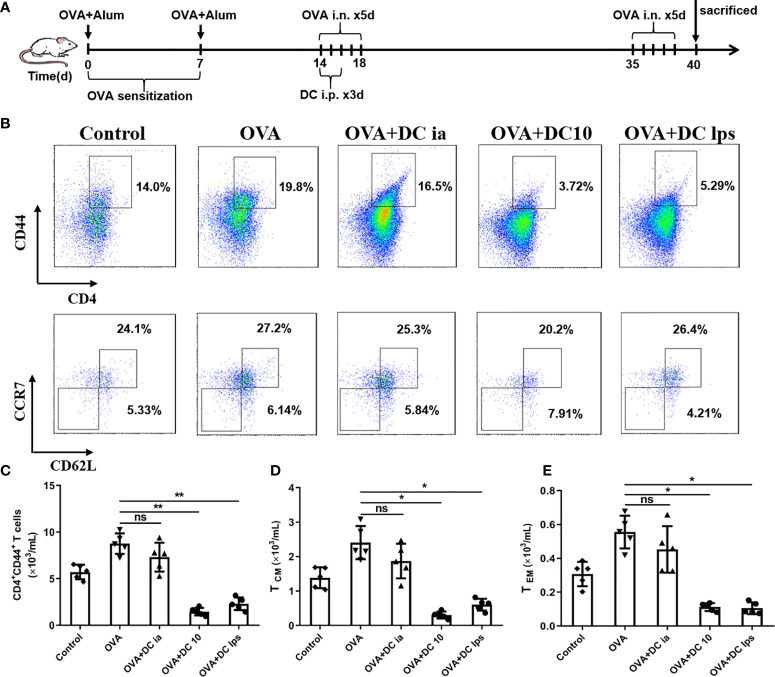
Adoptive transfer of DClps reduced the number of pulmonary memory CD4^+^ T cells. **(A)** Schematic of the OVA-induced asthmatic mouse model. The general experimental design is described in the main text. **(B)** Analysis of pulmonary memory CD4^+^CD44^+^ T cells by FCM. Central memory CD4^+^ T cells (T_CM_) were determined as memory CD4^+^CD44^+^CD62^+^CCR7^+^ T cells. Effector memory CD4^+^ T cells (T_EM_) were determined as memory CD4^+^CD44^+^CD62L^−^CCR7^−^ T cells. **(C)** Cell numbers of memory CD4^+^CD44^+^ T cells sorted by FCM in each group. **(D, E)** Cell numbers of T_CM_ and T_EM_ sorted by FCM in each group. The columns and error bars represent the mean and SEM. (*P < 0.05, **P < 0.01, ANOVA with Tukey’s *post hoc* analysis). The same experiment was repeated three times with similar results (n = 5 in each group).

It is widely believed that Th1/Th2 ratio imbalances may lead to the pathogenesis of allergic asthma, in which memory T cells play a crucial role in the capacity of initiating Th2 response under repeated allergen challenges (21, 22). Memory T cells can be divided into two distinct subsets, central memory T cells (T_CM_) and effector memory T cells (T_EM_), according to their homing characteristics and effector functions (23, 24). In order to assess whether DClps could suppress Th2 immune recall response in airways of treated mice, we sought to examine the number and function of memory CD4**^+^** T cells in lung tissues. We thus established asthma model mice and treated these mice with DClps, DC10 or DCia, as described above; however, instead of sacrificing the mice on day 19, we rechallenged the animals with OVA from days 35–39 (**Figure 4A**).

On day 40, mice were sacrificed and lungs were removed to obtain lung mononuclear cells. Pulmonary memory CD4**^+^**CD44**^+^** T cells were determined and sorted by FCM as mentioned above. We confirmed that both DC10 and DClps observably decreased pulmonary memory CD4**^+^**CD44**^+^** T cells, whereas the difference in DCia group was negligible compared to OVA group (**Figures 4B, C****)**. On the other hand, pulmonary memory CD4**^+^**CD44**^+^** T cells determined and sorted into two cell subpopulation: T_CM_ (CD4**^+^**CD44**^+^**CD62L**^+^**CCR7**^+^**) and T_EM_ (CD4**^+^**CD44**^+^**CD62L**^-^**CCR7**^-^**) by fluorescence-activated cell sorting (**Figure 4B**). Surprisingly, we saw no discernible difference in T_CM_ or T_EM_ cell numbers among mice treated with DClps, DC10 or DCia (**Figures 4B, D, E****)**.

### Adoptive Transfer of DClps Inhibited Pulmonary Memory CD4^+^ T Cell Proliferation

**Figure 5:**
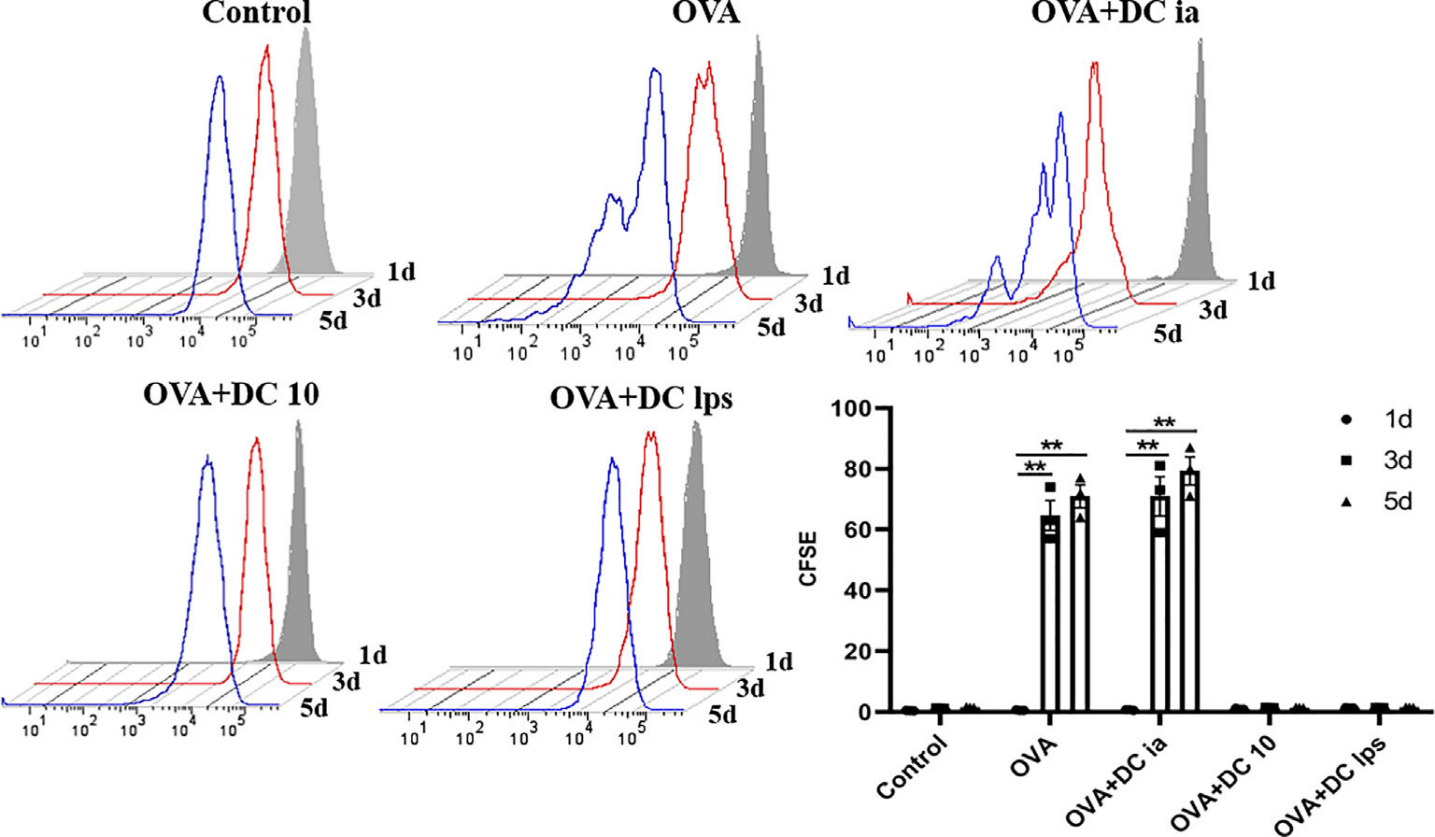
Adoptive transfer of DClps inhibited pulmonary memory CD4^+^ T cell proliferation. Pulmonary memory CD4^+^CD44^+^ T cells were sorted by FCM and then stained by CFSE. After MLR, the proliferation activity of memory CD4^+^CD44^+^ T cells was determined by FCM. The columns and error bars represent the mean and SEM. (**P < 0.01, ANOVA with Tukey’s *post hoc* analysis). The same experiment was repeated three times with similar results (n = 5 in each group).

To gain insight into this phenomenon, we assessed whether adoptive transfer of DClps affected the proliferation of pulmonary memory CD4**^+^** T cells with an MLR assay. The proliferation activity of sorted pulmonary memory CD4**^+^** CD44^+^ T cells was determined by CFSE method. Consistent with the above results, it was readily apparent the proliferation activity of pulmonary memory CD4**^+^** T cells in the DC10 and DClps group was obviously suppressed, compared with the DCia and OVA group, especially on days 3 and 5 (**Figure 5**).

### Adoptive Transfer of DClps Changed the Cytkoine Releasing Ability of Pulmonary Memory CD4^+^ T Cell

**Figure 6:**
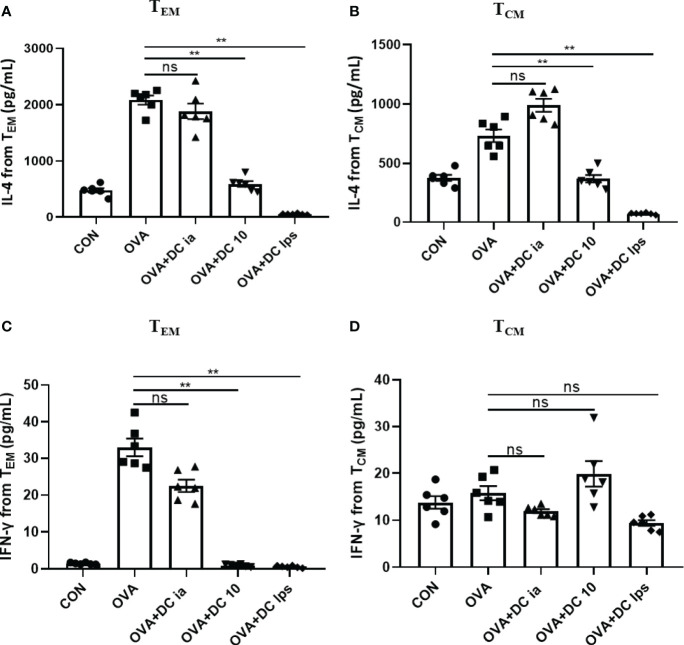
Adoptive transfer of DClps changed the cytokine releasing ability of pulmonary memory CD4^+^ T cell. **(A)** IL-4 released from pulmonary T_CM_ and T_EM_ determined by ELISA. **(B)** IFN-γ released from pulmonary T_CM_ and T_EM_ determined by ELISA. The columns and error bars represent the mean and SEM. (**P < 0.01, ns, no significant difference, ANOVA with Tukey’s *post hoc* analysis). The same experiment was repeated three times with similar results (n = 5 in each group).

Meanwhile, we collected the cell supernatant from the coculture system to assess IL-4 and IFN-γ secretion by memory CD4**^+^** T cells. We found that compared with cells in the OVA group, both T_EM_ and T_CM_ cells exhibited a notable decline in the secretion of IL-4 in the DClps and DC10 groups (**Figure 6A**), whereas no treatment effect was detectable in the DCia group (**Figure 6A**). Interestingly, in the DClps and DC10 groups, the level of IFN-γ dramatically reduced in only T_EM_ cells but not in T_CM_ cells (**Figure 6B**). Taken together, these data indicate that the adoptive transfer of DClps may inhibit the allergen recall response by suppressing memory CD4**^+^** T cells. Moreover, the reduction in memory CD4**^+^** T cells may well reduce the conversion of Th2 cells, which further results in the downregulation of Th2 cytokines and improves the Th1/Th2 imbalance in the immune response.

### Adoptive Transfer of DClps Increased the Number of Pulmonary Tregs in OVA-Induced Asthmatic Mice

**Figure 7:**
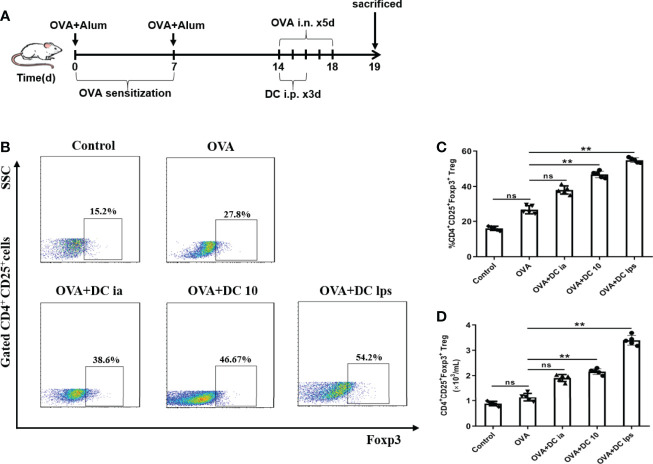
Adoptive transfer of DClps increased the number of pulmonary Tregs in OVA-induced asthmatic mice. **(A)** Schematic of the OVA-induced asthmatic mouse model. **(B)** Pulmonary Tregs were characterized as CD4^+^CD25^+^Foxp3^+^ cells by FCM. **(C)** Percentage of pulmonary Tregs determined by FCM. **(D)** Numbers of pulmonary Tregs determined by FCM. The columns and error bars represent the mean and SEM. (**P < 0.01, ns, no significant difference, ANOVA with Tukey’s *post hoc* analysis). The same experiment was repeated three times with similar results (n = 5 in each group).

Recent studies have demonstrated that DC10 treatment is associated with the conversion of Th2 effector T (Teff) cells into CD4**^+^**CD25**^+^**Foxp3**^+^** Tregs in the lungs of treated mice, and these Tregs are probably involved in inflammation regulation (25). Hence, we sought to examine whether DClps treatment increased the number of pulmonary Tregs in our asthma models. Lung mononuclear cells of asthmatic mice were obtained on day 19 and the expression of CD4, CD25, and Foxp3 were evaluated by FCM (**Figures 7A, B**). In line with previous studies that LPS-treated BMDCs cause immune tolerance through modulating CD4**^+^** Tregs (26), both DClps and DC10 treatment increased the number of pulmonary CD4**^+^**CD25**^+^**Foxp3**^+^** Tregs in the treated mice. However, we saw negligible differences in the number of Tregs between OVA group and DCia group (**Figures 7C, D****)**. Hence, our results suggest that adoptive transfer of DClps may alleviate airway inflammation by increasing the number of pulmonary Tregs in OVA-induced asthmatic mice.

### Adoptive Transfer of DClps Decreased the Phosphorylation Level of STAT6 in OVA-Induced Asthmatic Mice

**Figure 8:**
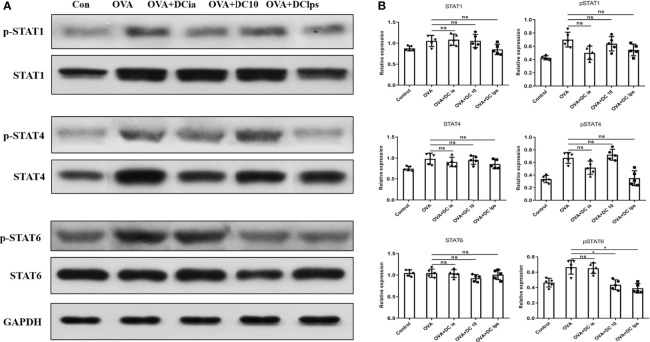
Adoptive transfer of DClps decreased the phosphorylation level of STAT6 in OVA-induced asthmatic mice. **(A)** Expression and phosphorylation of STAT determined by WB. **(B)** Quantification of STAT expression and phosphorylation, normalized to GAPDH, shown as relative expression. The columns and error bars represent the mean and SEM. (*P < 0.05, ns, no significant difference, ANOVA with Tukey’s *post hoc* analysis). The same experiment was repeated three times with similar results (n = 5 in each group).

Having shown that DClps treatment might be associated with the alleviation of airway inflammation in treated mice, we explored the potential mechanisms for this phenomenon. The STAT1 and STAT4 pathways are considered vital for Th1 differentiation, while the IL-4/IL-13/STAT-6 pathway has been confirmed to be the major modulator of Th2 differentiation in asthma pathophysiology (27, 28). To verify whether DClps modulate the airway inflammation of asthmatic mice by affecting the STATs pathways, we measured the total protein level and phosphorylation levels of STAT1, STAT4, and STAT6 in lung homogenates by WB analysis. We found that in asthmatic mice treated with DClps and DC10, the phosphorylation level of STAT6 protein was remarkably decreased, whereas the phosphorylation level of STAT1 and STAT4 protein merely showed a moderate downward trend that was not statistically significant (**Figures 8A, B****)**. We also noted that there were no recognizable changes observed in DCia-treated mice. On the basis of the above findings, we concluded that STAT6 signaling may be involved in the regulation of airway inflammation in asthmatic mice treated with DClps (**Figure 8**).

## Discussion

In this study, we generated diverse DCs from the bone marrow of wild-type mice that stimulated with LPS or IL-10, and delivered these cells to OVA-sensitized/challenged mice. The impact of DCia, DC10 or DClps on lung allergic inflammation, pulmonary memory CD4**^+^** T cells and pulmonary Tregs was determined. Apart from the downregulation of costimulatory molecules and proinflammatory cytokines, the upregulation of IL-10 and TGF-β secreted from specific DC can facilitate tolerance (8). Our results show that DClps were phenotypically similar to previously reported tolDC (29, 30), which exhibited signally reduced levels of MHCII, CD80, CD86 and IL-12, and enhanced levels of inhibitory cytokines (IL-10 and TGF-β) compared to DCia. Our observations further confirm that DC expressing low levels of costimulatory molecules and high levels of anti-inflammatory cytokines might take on a decided tolerance advantage.

*In vivo* studies showed intravenous transfer of DC10 reportedly markedly impeded airway allergic inflammation in murine models of OVA-induced asthma (31, 32). Moreover, the intravenous delivery of LPS-induced tolDC notably prohibited EAE development in a mouse model (26), and Zheng et al. proved that LPS-activated plasmacytoid DC effectively alleviated experimental chronic kidney disease (33). Intriguingly, there is conflicting evidence the intranasal administration of DClps producing high levels of IL-10 enhanced airway inflammation (34), and the intravenous infusion of DC10 failed to reverse the asthma phenotype in sensitized mice (35). Whether DClps can be used to ameliorate allergic asthma has not been completely explored. The salient discoveries from our study show for the first time that the intraperitoneal transfer of DClps after OVA challenge considerably alleviated the airway inflammatory cell infiltration and eosinophilia, suggesting that the delivery routes of DClps might influence their protective effect in treated animals. On the other hand, the injection of DCia had negligible regulatory effects on airway inflammation; therefore, our data clearly indicate the pivotal functional differences between LPS-stimulated and immature DC *in vivo*.

When DClps migrate to the lung has not been clarified, nor had it been assessed whether DClps stably occupy the lung *in vivo*. Huang et al. previously reported that DC10 that were intraperitoneally delivered predominantly migrated to the lungs over 1 week and were visibly reduced within 2 weeks, which corresponded to the time span over which lung inflammation improved (12). On the other side, DC10 treatment have been shown to induce progressive decreases in Th2 responses (cytokines, eosinophilia, and IgE) to levels near background over the next 2–5 weeks (36). Others have confirmed that LPS activation is required for migratory activity and antigen presentation by tolDC generated with dexamethasone (37). Similarly, our DClps tracking data showed that injected cells well migrated to lungs but were active for only 1 week. It remains unexplored why DClps in this study only affected tolerance over 7 days while others have observed impacts over 2–5 weeks (12, 36), but the findings were not contradictory since the modeling time of animals and some treatment conditions differed in these studies.

Allergen-specific Th2 memory cells have been viewed as fundamental for the development of allergic asthma in both human and animal models (22), and DC regulate the generation of memory T cells at each stage *via* the interplay between these cells (38). T_CM_ has the characteristics of self-renewal ability and long-term memory, which can proliferate after antigen restimulation; while the proliferation ability of T_EM_ is lower than T_CM_, whose function is similar to effector T cell (24). We thus rechallenged the animals with OVA and assessed pulmonary memory CD4**^+^** T cells, as described above. Of note, we showed that DClps and DC10, but not DCia, markedly decreased the number and proliferation activity of CD4**^+^**CD44^+^ memory T cells. Similarly, our MLR assay data confirm that IL-4 secretion was reduced in both T_CM_ and T_EM_ cells from DClps-treated mice, but IFN-γ was reduced only in T_EM_ cells, suggesting that adoptive transfer of DClps or DC10 may alleviate Th2 inflammation by suppressing the immune recall response. There was no difference in the cell numbers of T_CM_ and T_EM_ among the groups. It has been reported that the immune characteristics of CCR7**^+^**T_CM_ were significantly correlated with asthma severity and IgE levels, and the proportion of CCR7**^+^**T_CM_ in peripheral blood mononuclear cells (PBMC) in patients with allergic asthma was significantly higher than that in patients without allergic asthma (22). However, the airway immune microenvironment of asthma is complex and ample experimental evidence supports that T_CM_ and T_EM_ cells can be converted to the other cell type in many conditions (21, 23), hence, the exact mutual proportion of T_CM_ and T_EM_ need to be further investigated in the future.

We showed that the number of pulmonary Tregs increased in the DClps and DC10 group, which is consistent with previous studies that LPS-treated BMDCs induce immune tolerance by activating CD4**^+^** Tregs (24). Additionally, there is evidence showing that regulatory DC prohibited both antigen-specific IgE production and allergic responses *via* the expansion of CD4**^+^**CD25**^+^**Foxp3**^+^**Tregs (14), and Tregs were shown to remarkably increase in kidney-draining lymph nodes and kidneys after the adoptive transfer of DClps (33). Nevertheless, it has been reported that DC10 treatment ex vivo and *in vivo* did not augment the number of CD4**^+^**CD25**^+^**Foxp3**^+^** T cells but rather contributed to their activation (20, 25, 36). Besides, Zhou et al. demonstrated that LPS-stimulated DC cannot modulate Treg-associated molecules on CD4**^+^** T cells such as CD25 and Foxp3 *ex vivo* (39). Thus, wherein we were unable to unequivocally conclude that DClps have an ignorable impact on the number of CD4**^+^**CD25**^+^**Foxp3**^+^**Tregs in different diseases.

DC determines the differentiation of distinct T cell subsets and complex network of environmental signals and intrinsic cellular mechanisms affect DC’s tolerogenicity. Signal transducer and activator of transcription (STAT) is crucial in cytokines-induced cell responses, for example, IFN-γ induced STAT1 phosphorylation and IL-12 induced STAT4 phosphorylation can promote Th1 differentiation, whereas IL-4 induced STAT6 phosphorylation promotes Th2 differentiation. Medoff et al. demonstrated that STAT6 in BMDC was sufficient for the production of C-C motif chemokine ligand such as CCL17, CCL22, which are critical for Th2 lymphocyte recruitment to allergic airways (40). In present study, there is a trend of diminished expression of p-STAT4 in DClps group compared to OVA group, but it did not reach statistical significance (p >0.05). p-STAT6 in DClps and DC10 groups were obviously decreased compared to OVA group, which indicate DClps adoptive transfer reversed OVA-sensitized airway inflammation by inhibiting Th2-mediated inflammation.

In short, BMDC stimulated with LPS exhibited a tolerogenic phenotype. The intraperitoneal transfer of DClps inhibited the development of Th2 allergic responses by increasing Tregs, suppressing CD4**^+^** memory T cells and decreasing STAT6 phosphorylation level in OVA-induced asthma models. Further insight into the molecular mechanism and optimal process underlying the DClps-mediated immune tolerance should be investigated in the future.

## Data Availability Statement

The raw data supporting the conclusions of this article will be made available by the authors, without undue reservation.

## Ethics Statement

The animal study was reviewed and approved by Animal Care and Use Committee at Zhongshan Hospital, Fudan University (Shanghai, China).

## Author Contributions

ZC and MJ conceived and designed the study. ZM, YZ, and TZ performed the biological experiments. TZ, BC, and RM performed the statistical analysis. YZ, MJ, and ZC wrote and modified the paper. All authors contributed to the article and approved the submitted version.

## Funding

This work was supported by the National Natural Science Foundation of China (81470211 and 81970023 to ZC, 81970060 to MJ), the Shanghai Health Committee (201840288), the Shanghai Respiratory Research Institute and Yang Scientists Training Program of Zhongshan Hospital, the Shanghai Top-Priority Clinical Key Disciplines Construction Project (2017ZZ02013), and the Shanghai Municipal Key Clinical Specialty (shslczdzk02201).

## Conflict of Interest

The authors declare that the research was conducted in the absence of any commercial or financial relationships that could be construed as a potential conflict of interest.

## Supplementary Material

Supplementary Figure 1 Generation and characterization of bone marrow derived dendritic cells (BMDCs). **(A)** BM cells were cultured in RMPI-1640 medium with 10% FCS and 20ng/mL GM-CSF for 9 days. After incubation with PBS, 50ng/mL IL-10 or 10ng/mL LPS for 24h respectively, cells were stimulated with 1μM OVA for 2h, and DC ia, DC 10 and DC lps were collected. **(B)** BMDCs were first gated on CD11c and MHCII cells, and then the expression of CD80 and CD86 evaluated on CD11c^+^MHCII^+^ cells by FCM. Negative control was shown in upper panel.

Supplementary Figure 2 Central and effector memory T cells were first gated on CD4 and CD44 cells, and then the expression of CD62 and CCR7 evaluated on CD4^+^CD44^+^ cells by FCM. Central memory CD4^+^ T cells (T_CM_) were determined as CD4^+^CD44^+^CD62^+^CCR7^+^ memory T cells. Effector memory CD4^+^ T cells (T_EM_) were determined as CD4^+^CD44^+^CD62L^-^ CCR7^-^ memory T cells.

Supplementary Figure 3 Gating strategy of CD4^+^CD25^+^ Foxp3^+^ Tregs were shown in the representative FCM dot plots. Pulmonary Tregs were first gated on CD4 and CD25 cells, and then the expression of Foxp3 evaluated on CD4^+^CD25^+^ cells by FCM.

Supplementary Figure 4 Representative Masson staining of airway and parenchyma (scale bar = 100μm, Masson staining showed that collagen fibers appeared blue and muscle fibers appeared red). Percentage of the blue collagen-positive area in airway in each group. The columns and error bars represent the mean and SEM. (ns = no significant difference, ANOVA with Tukey’s *post hoc* analysis). The same experiment was repeated 3 times with similar results (n = 5 in each group).

## Abbreviations

DC, bone marrow-derived dendritic cells; DClps, LPS-activated BMDC; tolDC, tolerogenic dendritic cells; Tregs, regulatory T cells; DCia, immature DC; DC10, IL-10-induced DC; T_CM_, central memory T cells; T_EM_, effector memory T cells.
