# Supplementary figures and images for: Lipopolysaccharide-Activated Bone Marrow-Derived Dendritic Cells Suppress Allergic Airway Inflammation by Ameliorating the Immune Microenvironment
Source: Front Immunol. 2021 May 19;12:595369. doi: 10.3389/fimmu.2021.595369 (PMC8171252; doi:10.3389/fimmu.2021.595369)

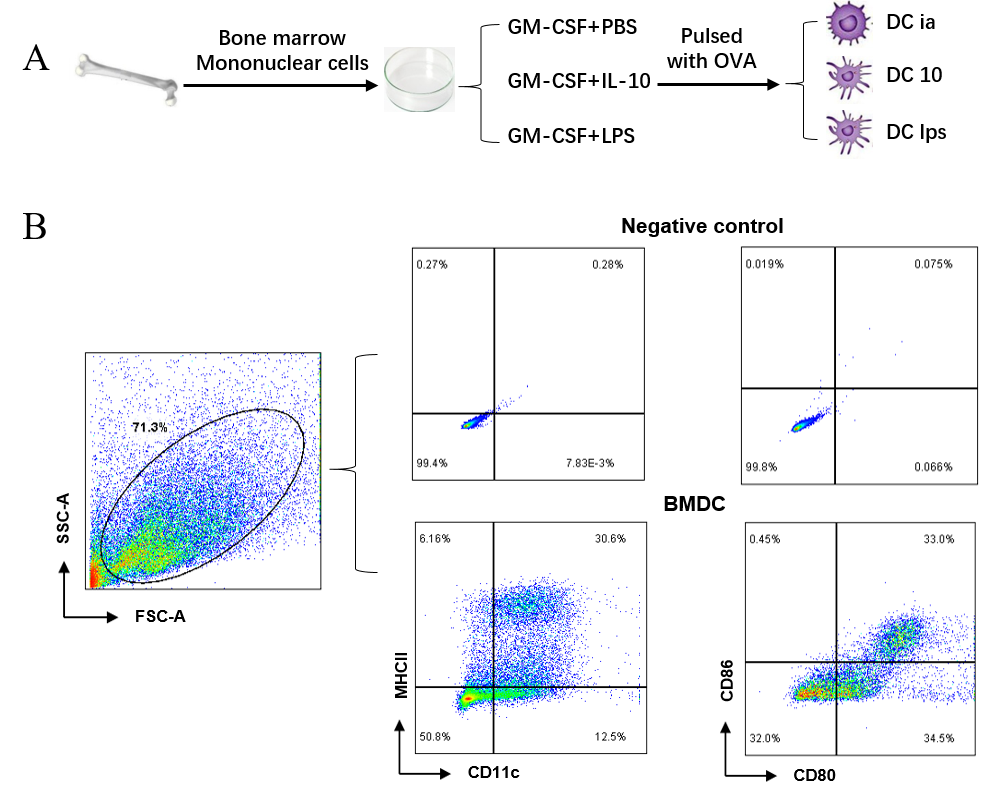

Supplement: Supplementary Figure 1 — Generation and characterization of bone marrow derived dendritic cells (BMDCs). (A) BM cells were cultured in RMPI-1640 medium with 10% FCS and 20ng/mL GM-CSF for 9 days. After incubation with PBS, 50ng/mL IL-10 or 10ng/mL LPS for 24h respectively, cells were stimulated with 1μM OVA for 2h, and DC ia, DC 10 and DC lps were collected. (B) BMDCs were first gated on CD11c and MHCII cells, and then the expression of CD80 and CD86 evaluated on CD11c+MHCII+ cells by FCM. Negative control was shown in upper panel. [file Image_1.tif]

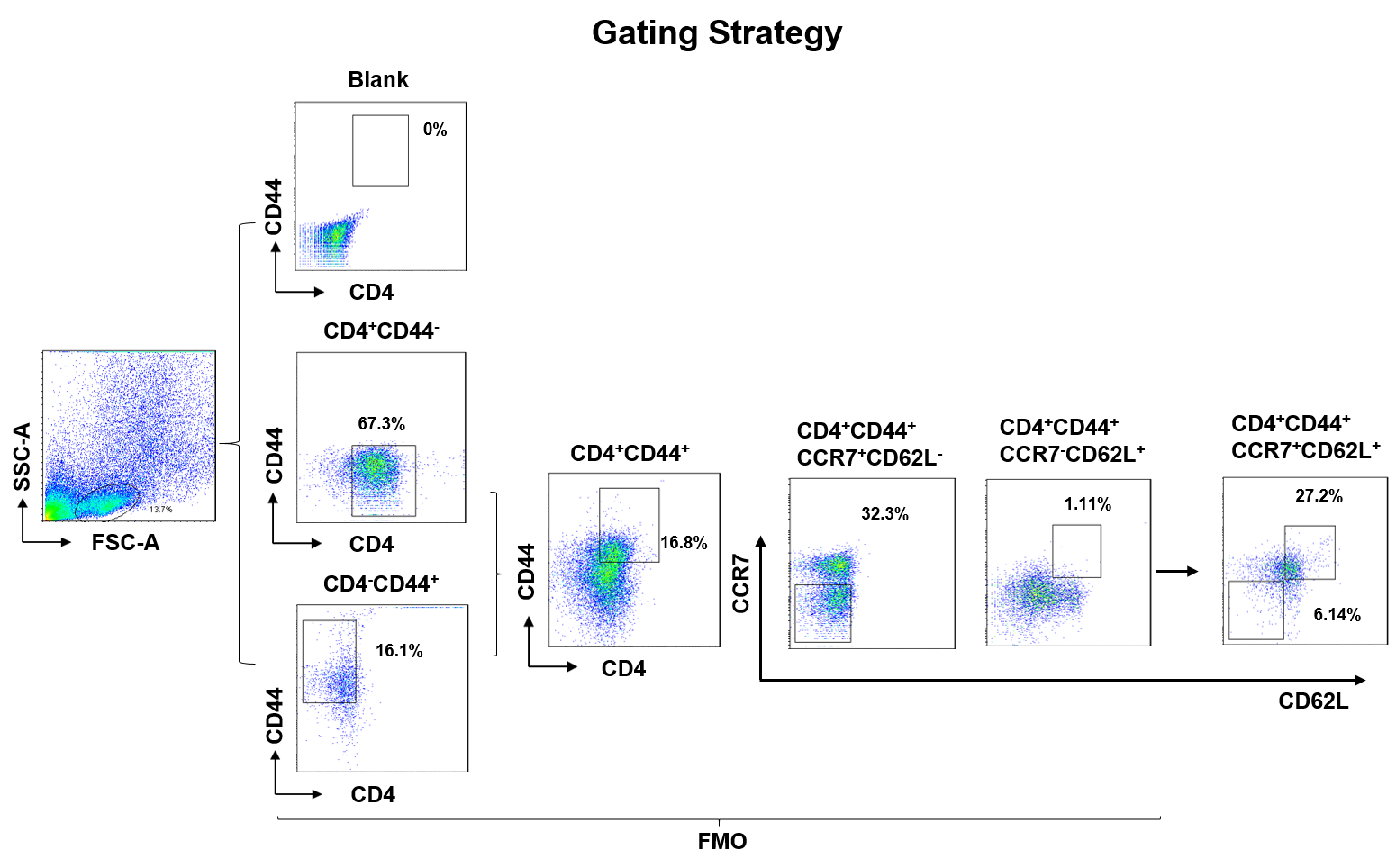

Supplement: Supplementary Figure 2 — Central and effector memory T cells were first gated on CD4 and CD44 cells, and then the expression of CD62 and CCR7 evaluated on CD4+CD44+ cells by FCM. Central memory CD4+ T cells (TCM) were determined as CD4+CD44+CD62+CCR7+ memory T cells. Effector memory CD4+ T cells (TEM) were determined as CD4+CD44+CD62L- CCR7- memory T cells. [file Image_2.tif]

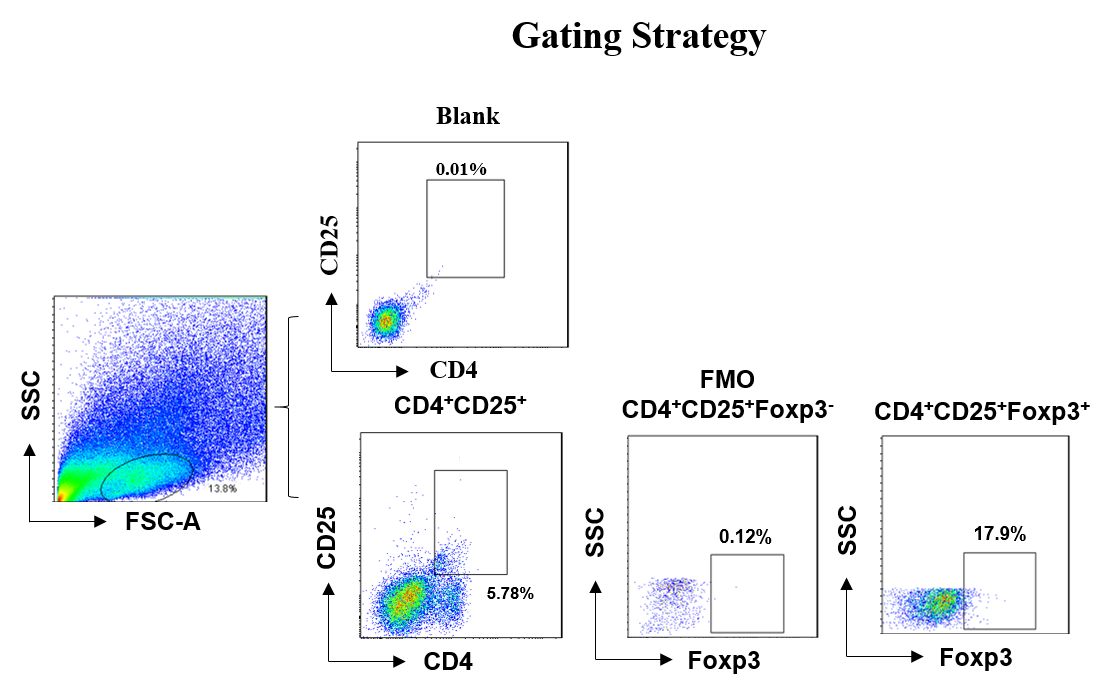

Supplement: Supplementary Figure 3 — Gating strategy of CD4+CD25+ Foxp3+ Tregs were shown in the representative FCM dot plots. Pulmonary Tregs were first gated on CD4 and CD25 cells, and then the expression of Foxp3 evaluated on CD4+CD25+ cells by FCM. [file Image_3.tif]

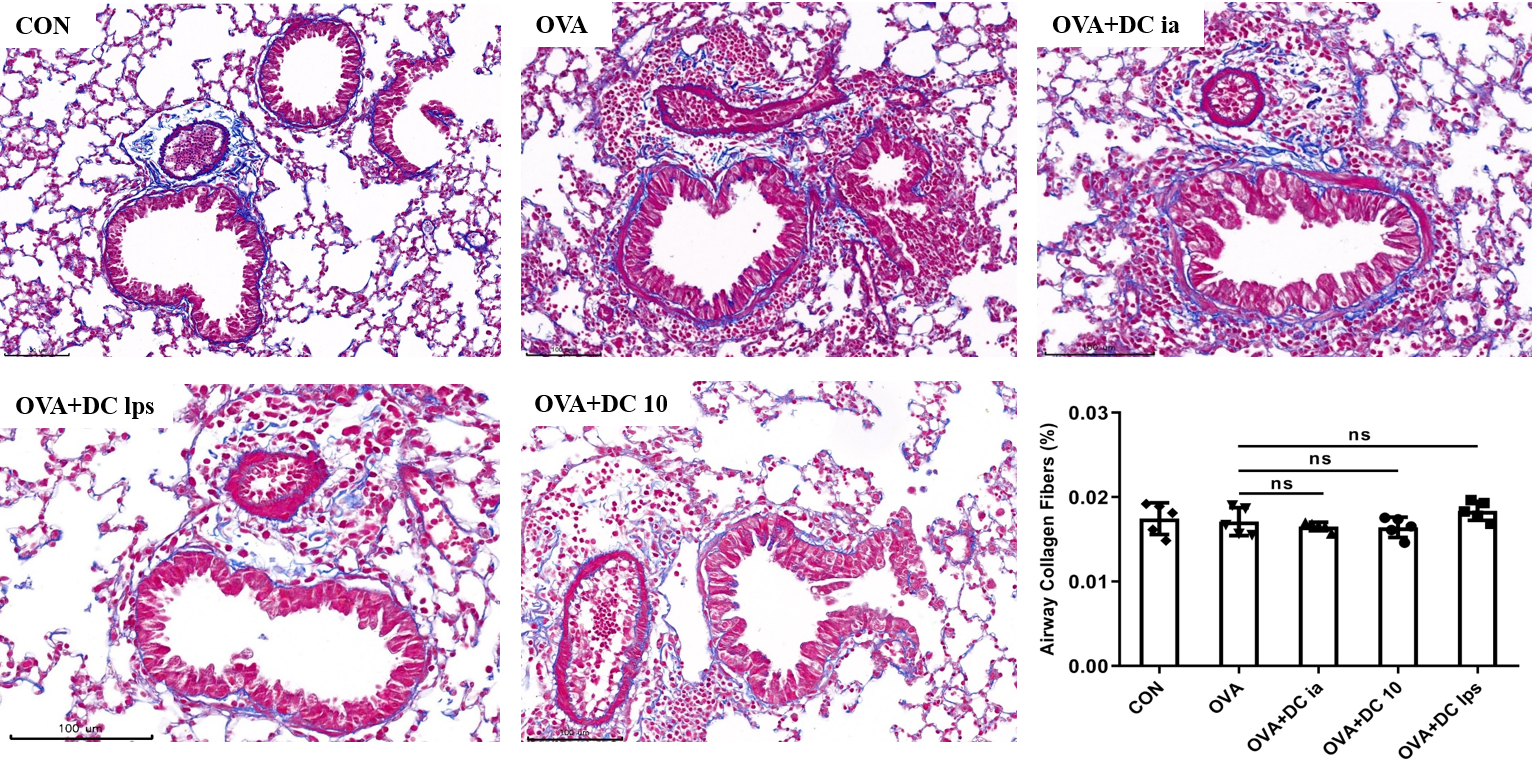

Supplement: Supplementary Figure 4 — Representative Masson staining of airway and parenchyma (scale bar = 100μm, Masson staining showed that collagen fibers appeared blue and muscle fibers appeared red). Percentage of the blue collagen-positive area in airway in each group. The columns and error bars represent the mean and SEM. (ns = no significant difference, ANOVA with Tukey’s post hoc analysis). The same experiment was repeated 3 times with similar results (n = 5 in each group). [file Image_4.tif]
